# ESSD–ESGAR best practice position statements on the technical performance of videofluoroscopic swallowing studies in adult patients with swallowing disorders

**DOI:** 10.1007/s00330-024-11241-1

**Published:** 2024-12-05

**Authors:** Martina Scharitzer, Wolfgang Schima, Margaret Walshe, Eric Verin, Stefano Doratiotto, Olle Ekberg, Daniele Farneti, Peter Pokieser, Emilio Quaia, Virginie Woisard, Ekaterini Xinou, Renée Speyer

**Affiliations:** 1 Department of Biomedical Imaging and Image-Guided Therapy, Medical University of Vienna, Vienna, Austria; 2 Department of Diagnostic and Interventional Radiology, Goettlicher Heiland Krankenhaus, Vienna, Austria; 3 Department of Diagnostic and Interventional Radiology, Barmherzige Schwestern Krankenhaus, Vienna, Austria; 4 Department of Diagnostic and Interventional Radiology, Sankt Josef Krankenhaus, Vienna, Austria; 5 Department of Clinical Speech and Language Studies, Trinity College Dublin, University of Dublin, Dublin, Ireland; 6 Department of Pulmonary Rehabilitation, UNIROUEN, Normandie University, Rouen, France; 7 Department of Diagnostic and Interventional Radiology, Ca‘ Foncello Hospital, Treviso, Italy; 8 Division of Medical Radiology, Department of Translational Medicine, Lund University, Skåne University Hospital, Malmö, Sweden; 9 Audiologic Phoniatric Service, ENT Department AUSL Romagna, Infermi Hospital, Rimini, Italy; 10 Teaching Center, Medical University of Vienna, Vienna, Austria; 11 Radiology Department, Padova University Hospital, University of Padova, Padova, Italy; 12 Voice and Deglutition Unit, Department of Otorhinolaryngology and Head and Neck Surgery, Larrey Hospital, University Hospital of Toulouse, Toulouse, France; 13 Radiology Department, Theagenio Cancer Hospital, Thessaloniki, Greece; 14 Department Special Needs Education, University of Oslo, Oslo, Norway; 15 MILO Foundation, Centre for Augmentative and Alternative Communication, Schijndel, The Netherlands; 16 Curtin School of Allied Health, Curtin University, Perth, WA Australia; 17 Department of Otorhinolaryngology and Head and Neck Surgery, Leiden University Medical Centre, Leiden, The Netherlands

**Keywords:** Fluoroscopy, Deglutition disorders, Dysphagia, Barium, Consensus development

## Abstract

**Objectives:**

Videofluoroscopic swallowing studies (VFSS) remain the gold standard for the instrumental assessment of oropharyngeal swallowing disorders alongside flexible endoscopic evaluation of swallowing (FEES), requiring a high standard of quality and correct implementation. The current best practice position statements aim to guide the clinical practice of VFSS in individuals experiencing swallowing disorders.

**Materials and methods:**

An international expert consensus panel with expertise in oropharyngeal dysphagia, comprised of radiologists, speech-language therapists, otolaryngologists, and other professionals in the field, convened by the European Society of Swallowing Disorders (ESSD) and the European Society of Gastrointestinal and Abdominal Radiology (ESGAR), developed best practice position statements. They were established using an online Delphi methodology involving an online panel discussion and item preparation and three consecutive rounds. Consensus was reached when ≥ 80% of the participants agreed on a specific recommendation.

**Results:**

Eighteen best practice position statements were formulated, thereby establishing standard recommendations on the technical performance of VFSS. They cover VFSS planning, correct implementation, documentation, radiation protection, equipment and maintenance, and education and training.

**Conclusion:**

These position statements summarise the panel’s deliberations and recommendations in performing VFSS, representing the agreed consensus of experts from ESSD and ESGAR. They provide a structured framework for optimising and standardising the performance of VFSS in patients with swallowing disorders.

**Key Points:**

***Question*** *Significant regional and national differences in clinical practice when performing VFSS highlight the need for interdisciplinary recommendations to optimise patient care*.

***Findings*** *Eighteen statements were developed by representatives of the ESSD and the ESGAR*.

***Clinical relevance*** *These best practice position statements on the technical performance of VFSS may serve as a basis for standardising the procedure and ensuring high-quality service*.

## Introduction

The videofluoroscopic swallowing study (VFSS), also referred to as the modified barium swallow, is a dynamic fluoroscopic examination used to assess swallowing biomechanics in patients of all ages. Since it was introduced almost 60 years ago [1], patients are referred to the radiology department to provide real-time visualisation of the passage of the bolus in relation to the movement of oral, pharyngeal, laryngeal, and oesophageal structures involved in the swallowing process. A VFSS serves diagnostic and therapy-guiding purposes by evaluating morphological pathologies and abnormal swallow function in a single investigation. It plays a crucial role in identifying the underlying cause of dysphagia and determining the most appropriate management strategies including suitable consistencies for oral intake and the impact of compensatory therapeutic manoeuvres for individual patient care.

Despite a global decline in the utilisation of fluoroscopic procedures, the demand for VFSS services is likely to persist given its clinical significance in diagnosing and managing patients with swallowing disorders due to its unique ability to depict all phases of the swallowing process. This need is reinforced by the increasingly ageing population. Thus, the use of VFSS has experienced a more than 20% increase in the last two decades [2].

The VFSS is a highly operator-dependent and complex procedure and can show significant variations in the clinical approach, implementation, and interpretation. Differences in education and training of allied health professionals lead to variations in VFSS practices within and between institutions at national and international levels. This includes patient referral criteria, the use of contrast agents, technical basics, and the selection of professionals performing the examination. Different approaches are also influenced by equipment availability and national regulations. The shift from image intensifiers to flat-panel fluoroscopy and picture archiving and communication systems (PACS) impacts the effectiveness of VFSS. Changes in the working environment have led to an expansion of the traditional roles of healthcare professionals performing VFSS, with non-radiologists increasingly moving into positions of responsibility. A survey of speech-language therapists in the United Kingdom has revealed a lack of standardisation of clinical practice and knowledge in fundamental areas of implementation of VFSS [3]. In a systematic review, published in 2019, Boaden et al [4] identified seven clinical practice guidelines worldwide with significant variance regarding the professional content and recommendations provided.

The notably significant discrepancies in clinical practice at various stages of the examination raise concerns. To ensure standardised practice for optimal patient care, clinicians involved in VFSS require methodologically sound recommendations which summarise the most reliable evidence for clinical practice and improve the use of limited healthcare resources. Our objective is to establish international European best practice position statements through the cooperation of the European Society of Swallowing Disorders (ESSD) and the European Society of Gastrointestinal and Abdominal Radiology (ESGAR) representatives to prevent unwarranted variation in the conduct of VFSSs that may negatively influence patient care. These position statements on best practices are not prescriptive guidelines. They apply to adults presenting with dysphagia and referred to VFSS. They may not be universally applicable, and their interpretation should consider specific clinical scenarios and resource availability.

## Material and methods

A multidisciplinary expert panel was selected by ESSD and ESGAR society with an equal number of representatives (*n* = 6 each) based on their expertise and achievements within the field. The panel collectively represented abdominal radiology (*n* = 7), speech-language therapy (*n* = 2), otolaryngology (*n* = 2), and physical medicine and rehabilitation (*n* = 1). Representatives comprised society members of Austria (*n* = 3), France (*n* = 2), Italy (*n* = 3), Greece (*n* = 1), Ireland (*n* = 1), Norway (*n* = 1), and Sweden (*n* = 1). The project was divided into three working groups covering the following topics: “Indications, contraindications and equipment specifications”, “contrast media and radiation safety” and “investigation specifications”. Working group leadership was balanced between ESSD and ESGAR representatives.

**Fig. 1:**
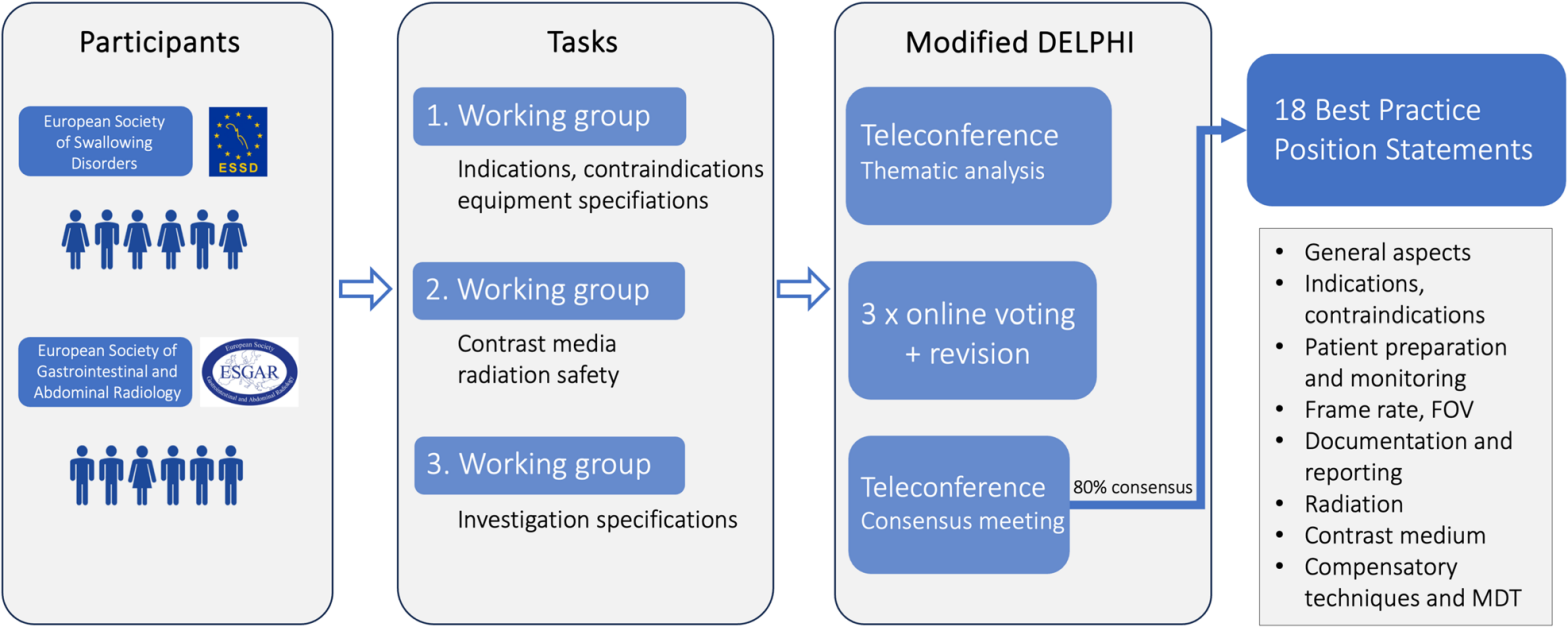
Overview of the modified Delphi study. FOV, field of view; MDT, multidisciplinary team

Best practice position statements were developed by following a modified Delphi process as described below. Each working group conducted literature research on their specific topics using PubMed covering articles without temporal constraints, in addition to referring to their own resources. Utilising these literature findings, each working group formulated best practice position statements accompanied by corresponding supporting text. The review of position statements occurred through a series of three online voting rounds using a computer-assisted web interviewing system, i.e. Google Forms (Google LLC) with the possibility to comment on each statement by all participants. In between the voting rounds, the statements were revised by the working groups according to the input and feedback from all panellists. Despite the panellists voting independently and anonymously, the voting rounds of the modified Delphi process did not ensure anonymity to the organiser. She maintained the anonymity of the votes and accompanying comments throughout and after the voting rounds. After the third online voting round, a final web-based video conference took place in November 2023 to discuss and vote on the updated statements. A consensus was defined a priori as an agreement between ≥ 80% of participants (Fig. 1). The project leaders edited the final manuscript, which was subsequently circulated among and approved by the consensus group participants. Next, it was submitted to the ESSD and ESGAR boards for approval. It is recommended to read the best practice position statements in conjunction with their corresponding supporting text rather than in isolation.

### Best practice position statements

#### General aspects

##### Best practice position statement 1

The request for a VFSS should include sufficient information to justify the medical necessity of the examination. It should be initiated by a physician or other licensed healthcare provider following local and national policies and procedures.

Videofluoroscopy carries some radiation risk, as well as aspiration risk with the ingestion of an oral contrast medium. The request should consider the medical risk to the patient. The referring physician or other licensed healthcare provider, who is a specialist in the area of swallowing assessment is best placed to consider this risk.

Local and national policies regarding the referral procedure should be in place. It is recognised that policies and procedures will vary nationally and internationally [5–7].

##### Best practice position statement 2

The patient’s eligibility must be determined based on medical history, clinical assessment, and review of pertinent information that may impact the procedure before starting the investigation. Knowledge of the medical history prior to the examination is relevant in order to individually tailor the examination technique to the patient’s symptoms.

A careful case history is critical in formulating a clinical hypothesis regarding the aetiology of dysphagia, whether signs and symptoms of dysphagia are consistent with oropharyngeal or oesophageal dysphagia and providing information on the need for a VFSS in conjunction with or instead of other instrumental evaluations [8–11]. The signs and symptoms of dysphagia may help differentiate between oral, pharyngeal or pharyngo-oesophageal difficulties.

Pertinent medical information that may impact swallowing is important. This may include timing of medications [12, 13], oxygen requirements, and blood pressure status. Previous examinations should be reviewed to avoid unnecessary radiation exposure, address specific questions during the study, and allow for comparison between studies. The patient’s ability to complete the VFSS procedure will ideally be confirmed by the clinical swallowing examination (CSE) [14] although it is recognised that this is not always possible. The CSE should help confirm the suspicion or presence of oropharyngeal and/or oesophageal dysphagia and may modify the VFSS protocol. Patients, who are unable to manage oral trials due to cognitive impairment, will not be able to complete the VFSS, which requires the ingestion of oral contrast medium.

#### Indications and contraindications

##### Best practice position statement 3

Indications for a VFSS include the need for assessment of the oral preparatory, oral transit, pharyngeal, and/or oesophageal phases of swallowing in all patients with symptoms related to eating and drinking. These may be either subjectively reported or observed by a caregiver, and VFSS findings should have the potential to influence patient management. In some instances, VFSS may be performed as a “diagnostic” test accompanied by a “therapeutic-guiding” test, although there is considerable overlap between the two.

Patients with symptoms related to swallowing, whether self-reported by the patient, observed by a caregiver, or identified during a clinical swallowing examination, may be referred for VFSS [15]. These symptoms and clinical conditions may especially include dysphagia, suspicion of aspiration, sensation of a lump in the throat (globus sensation), respiratory disorder, feeding difficulties, diagnosed or suspected abnormalities along the upper gastrointestinal tract, the need to evaluate therapeutic manoeuvres or other management strategies, or a medical condition with a recognised high risk for swallowing disorders [16].

A diagnostic VFSS aims to clarify the clinical symptoms of a patient and evaluate the extent of impairment by evaluating the bolus flow in relation to structural movement, timing, and coordination throughout the oropharyngeal and oesophageal tract. For a therapeutic-guiding VFSS, various compensatory strategies, such as adjusting bolus volume or consistency and implementing postural or swallowing manoeuvres, are tested and documented using videofluoroscopy to assess their impact on swallowing efficiency and safety [4, 17]. Therapeutic-guiding studies should not be performed without prior diagnostic evaluation [18]. Nonetheless, both parts often intersect and are ideally combined into one examination.

Patients presenting with rapidly progressing obstruction for solids, weight loss, or other alarm symptoms should undergo endoscopy first as a priority before considering a VFSS. VFSS can serve as an initial assessment of swallowing function or as a repeated evaluation for patients whose swallowing capabilities are likely to change due to disease progression or improvement following an intervention. Additionally, VFSS may diagnose aspiration or residues, when a patient is unable or refuses to undergo transnasal endoscopy.

In patients with chronic obstructive pulmonary disease (COPD) or other pulmonary conditions often accompanied by dyspnoea and, in some cases, abnormal lung function associated with aspiration or gastro-oesophageal reflux, VFSS is used to evaluate the patient’s ability to safely meet nutritional requirements on oral intake and manage oral medications.

##### Best practice position statement 4

Contraindications include patients with an unstable medical condition, insufficient cognitive awareness to cooperate, inability to provide sufficient posture for imaging, or lack of possible therapeutic changes in the individual management plan.

There are very few contraindications for undergoing a VFSS. One requirement is that the patient should be sufficiently conscious to actively cooperate in the examination, particularly with the goal of being at least partially fed orally. The radiologic equipment should accommodate the patient’s physical function, and the patient should attain and maintain a suitable posture. It does not constitute a contraindication, when the patient is allergic to barium or other components of the contrast material. In this case, an alternative contrast material, such as non-ionic iodine agents, should be considered. Patients with fistulae, such as trachea-oesophageal, should not undergo an examination with barium but with non-ionic iodine. Relative contraindications include pregnancy, patients on ventilators and for whom portable ventilation is not possible, and if the patient is nil by mouth for reasons unrelated to swallowing dysfunction.

#### Patient preparation and monitoring

##### Best practice position statement 5

No special preparation is needed for VFSS.

The patient is examined with their dentures or other oral appliances in place so that their swallow is as normal as possible. In most cases, the nasogastric feeding tubes can be left in situ due to discomfort during reinsertion of tubes, since the presence of a nasogastric tube does not substantially alter videofluoroscopic findings [19, 20]. Metal objects within the field of view have to be removed before starting the investigation, including earrings, necklaces, eyeglasses, or hairpins. Fasting may be required depending on the patient’s symptoms, for example, to reduce pharyngeal or oesophageal residues.

##### Best practice position statement 6

Although no special monitoring is needed for patients undergoing videofluoroscopy, equipment for medical emergency response should be available.

Equipment for handling emergencies should be identified and available. Before starting the examination, patients at risk of immediate respiratory failure or severe aspiration, who require immediate treatment, should be identified. A suction device (in-wall vacuum system or portable suction unit) and pulse oximetry should be available in the fluoroscopy room and checked at regular intervals to ensure it is in good working condition. In accordance with local regulations and policies, it is mandatory to have medical personnel with proper training and current knowledge present to provide immediate assistance in cases where patients are choking and aspirating.

#### Frame rate, field of view

##### Best practice position statement 7

VFSS should be performed at a high spatial and temporal resolution with a minimum of 15 frames per second. The detector should capture a frame rate that corresponds to the pulse rate.

High spatial resolution can be obtained using both currently available detector technologies for fluoroscopy: flat panel detectors and the older technology of image intensifiers. Both types of equipment provide a high temporal resolution of 25 or 30 pulses per second. Time-dependent physiological components like aspiration, laryngeal hyoid movement [21], and dynamic morphological observations like webs or rings [22] often present for only fractions of a second, justifying the necessity for a high temporal resolution. The recommended pulse rate is at least 15 pulses per second, preferably with 25 or 30 pulses per second [21, 23]. The selection of a specific pulse rate should align with the corresponding frame rate of images recorded by the detector of the fluoroscopy unit.

Audio recording integrated into the fluoroscopy system can be useful for the evaluation of oropharyngeal dysphagia. Dose reference levels (DRLs) must be carefully balanced, considering the individual patient’s benefit. They must be documented and audited according to national regulations as agreed with the medical physics expert. X-ray equipment must adhere to national guidelines and be adaptable to a range of patients’ positioning requirements. Radiolucent seats may be necessary, and attention should be given to the patient’s height and position in relation to the X-ray machinery.

##### Best practice position statement 8

The lips, the oral cavity, the soft palate, the nasopharynx, the oropharynx, the hypopharynx, the larynx, and the upper oesophagus are the structures that need to be in the field of the oropharyngeal exam during VFSS.

The fluoroscopic image should be centred on the important area for evaluation (oral cavity/pharynx/upper oesophagus/airways) to provide the most accurate view of the structures that need to be examined, avoiding the lenses of the eye [18]. During the exam, it is possible to adapt the patient’s position in order to focus on a particular area.

The boundaries of the fluoroscopy field in the lateral view are the lips anteriorly, the nasopharynx superiorly, the cervical spinal column posteriorly and the cervical oesophagus inferiorly [24]. This comprehensive view enables the assessment of the various stages of swallowing at one time [25]. For the anterior–posterior (AP) views, the field of view is initially focused on the oral cavity so that the palate forms the superior border of the image and the vocal folds and tracheal column form the inferior border.

**Fig. 2:**
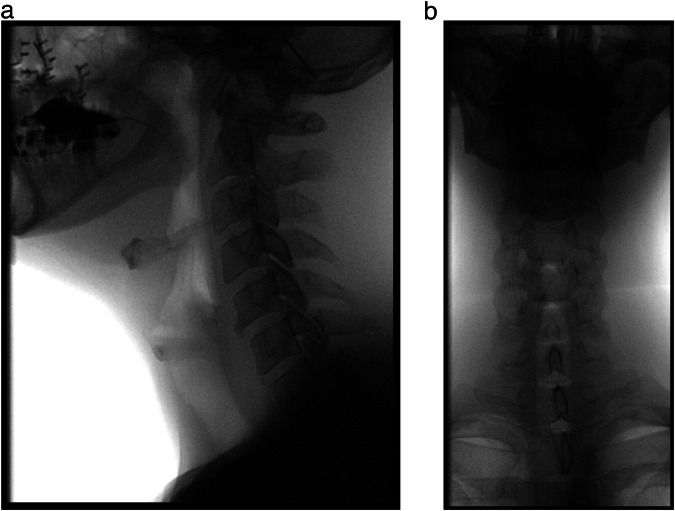
Field of view of a lateral and anteroposterior oropharyngeal VFSS sequence: female patient with oropharyngeal dysphagia after maxillofacial trauma and reconstruction. In the lateral view, (**a**) the boundaries of the fluoroscopy field encompass the lips, the nasopharynx, the cervical spinal column and the cervical oesophagus. For the anteroposterior view (**b**), the boundaries are superiorly the palate, inferiorly the cervical oesophagus and laterally the pharynx

To improve the quality of the image, the field is collimated bilaterally to the angles of the mandible [25]. In addition to image quality considerations, appropriate collimation also reduces the size of the radiation field, resulting in lower radiation dose. Therefore, collimation should always be employed to the extent that it does not compromise the visualisation of crucial anatomy (Fig. 2a, b).

##### Best practice position statement 9

Visualisation of the entire oesophageal phase with evaluation of the oesophageal clearance of bolus in the upright position is recommended to be routinely performed during VFSS, if feasible.

During VFSS, it is essential to evaluate both oropharyngeal swallowing and oesophageal clearance in the upright position. This is particularly important because localising the level of dysphagia can be challenging despite comprehensive history taking [26]. Up to one-third of patients reporting lower throat symptoms may have an oesophageal cause for dysphagia [27] that could remain undetected without the observation of the oesophageal phase during VFSS [28]. Surveying the oesophagus is also important because many patients experience multiphase swallow impairment, with concurrent oropharyngeal and oesophageal disorders [29, 30]. Therefore, evaluating oesophageal function should be a routine part of the assessment for all patients complaining of dysphagia or globus sensation when referred for VFSS [31, 32].

Observing the oesophageal clearance in the upright position during VFSS does not intend to assess oesophageal motility thoroughly [25], nor does it replace the air contrast barium oesophagram due to its limited sensitivity [33]. A well-established oesophageal screening protocol may facilitate timely referral for additional assessments [28, 29, 34]. Given its low sensitivity, if oesophageal screening is negative, but there is high clinical suspicion, a subsequent thorough oesophagram including investigation in the horizontal plane should be recommended. Additionally, manometry, or impedance-manometry could be considered [33].

#### Documentation and reporting

##### Best practice position statement 10

Fluoroscopy sequences must be captured and documented without significant quality loss, consistent with local legal requirements, in a local PACS to allow post-examination replay for analysis.

The recording of fluoroscopic sequences, along with their subsequent transfer and storage in the patient’s medical record, is essential for later frame-by-frame analysis, communication with intra- and extramural specialists in the multidisciplinary team, comparison for improvement or deterioration assessment, and avoidance of unnecessary repeat examinations. Video sequences, as well as spot radiographs or screenshots if deemed necessary during the investigation, should be stored in a PACS, preferably using the digital imaging and communications in medicine format, which is the digital image standard.

The equipment used should support continuous and frame-by-frame analysis, featuring an adjustable speed for forward and backward movement. Maintaining temporal and spatial resolution throughout the data storage process is important, ensuring compatibility between the fluoroscopy unit and the recording system. Additionally, the application of image compression standards should be approached cautiously to prevent any loss of resolution [35].

##### Best practice position statement 11

The report should include relevant aspects of the technical performance of the examination, patient positioning, the area covered by VFSS, the protocol used, limitations affecting diagnostic accuracy, and the required radiation dose.

The written report of a radiological investigation is the most critical communication between performing physicians, referring medical doctors, and other healthcare professionals [36, 37]. A comprehensive VFSS report should include a concise description of examination parameters, including the fluoroscopy equipment specifications, the frame rate/pulse rate used, and whether radiographs were obtained. Patient positioning, projections, and the specific area covered by fluoroscopy, along with compensatory strategies or rehabilitative techniques, should be clearly described.

The type, consistency, volume of contrast medium used and details regarding its administration, and feeding utilities are essential aspects of a report. Procedure-related instructions given to the patient should be documented. Any technical and patient-related features that may impact the accuracy of the interpretation need to be identified and documented. If accessible, the patient’s radiation dose should be included in the report.

The use of standardised template reports has demonstrated an improvement in the quality and comprehensiveness of reports within a centre [38]. However, their effectiveness relies on the use of commonly known terms and should be implemented according to local policies (Table 1).

**Table 1:** Key quality technical and procedural parameters that should be included in an adequate report

Pre-procedure features
Indication for referral, information from clinical history interview
Frame rate/pulse rate
Intra-procedure features
Patient positioning (standing, sitting, chair, …)
FOV, areas covered (oropharynx, lateral/frontal position, and oesophagus)
Number of trials tested, use of additional radiographs
Contrast medium: type, volumes, and consistencies used
Administration and feeding utilities of contrast medium
Description of compensatory strategies or rehabilitative techniques used
Technical and patient-related limitations affecting diagnostic accuracy
Post-procedure features
Image storage location
Radiation dose, if accessible

#### Radiation

##### Best practice position statement 12

The radiation dose of VFSS is an important issue, although it is, in general, lower than that of a neck CT examination. There is a wide range of radiation doses imparted during VFSS, depending on the number of swallows recorded, the length of video loops, and the projections. Total radiation dose is especially important in the clinical scenario of repeated studies. The thyroid gland is the most radiosensitive organ in oropharyngeal studies.

Regarding the fluoroscopy time during VFSS, a wide variation has been reported. Benfield et al reported a median fluoroscopy time of 3 min (range 0.7–10 min) in a UK-based survey [39], Chau et al found mean fluoroscopy times of 4.23 min [40], and Bonilha et al of 2.4 min [41], with a range of 23–387 s. Accordingly, the imparted radiation doses also show high variability in various studies on adults (dose area product 1.6–11 Gy.cm², for a mean effective dose of 0.2–0.85 mSv) [42–45]. The fluoroscopy time, and consequently the effective radiation dose, depends on the clinical indication of VFSS [46]. Fluoroscopy times are longer (and effective doses are higher) in studies on cerebrovascular accident patients than on nasopharyngeal cancer patients [47].

The radiation dose of VFSS is considerably lower than that described for neck CT examinations in adults (median effective dose, 1.76 mSv and 5 mSv, respectively [48, 49]. There is no linear correlation between fluoroscopy time at VFSS and radiation dose imparted, because the radiation dose per time unit depends on the anatomic region assessed (lower doses for pharyngeal studies vs oesophageal studies).

In pharyngeal studies, the thyroid gland is the most radiosensitive organ exposed [50]. In general, the radiation dose is lower in the lateral than in the frontal projection (AP to posterior–anterior [PA] radiation beam) [41]. The use of an AP vs PA projection depends on the technical set-up: a fluoroscopy unit with an under-table X-ray source provides a PA radiation beam with a considerably lower thyroid dose than with an AP beam [41].

##### Best practice position statement 13

Radiation protection of personnel and patients has to be provided during VFSS procedures according to the “as low as reasonably achievable principle” (ALARA). Limiting fluoroscopy time and use of appropriate collimation during fluoroscopy, increasing the distance between X-ray tubes, and supervising personnel and appropriate lead shielding of personnel are key issues.

For dose optimisation, fluoroscopy time and number of swallows recorded have to be kept at a minimum, which still allows a safe diagnosis. The use of a larger field-of-view (FOV) with appropriate collimation reduces the radiation dose to the patient in comparison with magnification modes (small FOV), at the expense of lower image resolution [51]. Patients, who are fit and without cognitive impairment should deliver the boluses themselves to reduce the radiation exposure to the personnel. Personnel supervising the examination should always wear lead aprons including thyroid shields and radiation-monitoring badges underneath the aprons (to measure the actual dose imparted to the personnel). Although these recommendations seem self-evident, in an Australian survey on speech-language therapists, only 76% indicated they would always wear lead thyroid shields and only 36% wear radiation-monitoring badges during the examination [52]. An Australian follow-up study indicated only minimal improvement in awareness (43% reported always wearing radiation-monitoring badges) [53].

Standing behind a transparent lead screen or installing a vertical lead-shielding device on the handrail of the fluoroscopy table (to reduce scatter radiation from the patient´s head and body to the personnel) may further decrease radiation [54].

#### Contrast medium

##### Best practice position statement 14

Low-density barium suspension (20–40% barium weight/volume of water) is used in VFSS to visualise the morphology and function from the oral cavity to the oesophagus. Water soluble iodinated contrast media are recommended if there is a risk of perforation or leakage of contrast media into the tissues. Non-ionic low-osmolar iodinated contrast media are indicated for patients with a clinical suspicion of aspiration.

Low-density barium (20–40% *w*/*v*) suspension is used in VFSS to visualise the morphology and function from the oral cavity to the oesophagus [16]. Although some studies did not report any adverse effect of barium sulfate on the respiratory system [55, 56], others showed impaired pulmonary function if a large amount of barium enters the airway [57–60]. Barium in the extravisceral tissues tends to persist and may cause local inflammation [57].

Low-osmolar nonionic iodine-based contrast media are indicated for patients with a clinical suspicion of aspiration, perforation or leakage of contrast material into the tissue [16, 57]. Low-osmolar nonionic iodine contrast media applied in VFSS include either monomeric (e.g., iohexol [640 mmol/kg at 300 mg iodine/mL)] or dimeric (iodixanol) for oral application [57].

Hyperosmolar ionic iodinated contrast media, namely amidotrizoate (also known as diatrizoate, 1500–2000 mmol/kg, most common brand name Gastrografin®), are contraindicated in patients with suspicion of aspiration [16]. There is reason for caution, if hyperosmolar iodine-containing agents are aspirated, as there may be a shift of fluid into the alveoli and interstitial spaces, disrupting gas exchange and causing the development of acute pulmonary oedema from the pulmonary tissues into the airway [57, 61, 62]. In animal studies, ionic iodine-containing contrast agents caused low inflammatory response [63, 64]. Nonionic contrast media did not cause any discernible inflammatory response in the lungs, suggesting it may be the safest contrast for VFSS [63].

##### Best practice position statement 15

In a diagnostic VFSS, the normal amount of a single sip for an adult patient is about 10–15 mL, if tolerated by the patient. Different bolus consistency is indicated to determine the effect of food consistency on swallowing and to assess stricture or solid-induced spasm or dysphagia.

In a diagnostic VFSS, the average sip size of a single swallow for thin liquids is 10–15 mL [18, 65]. However, a healthy adult can manage liquid boluses of up to 50 mL or more [18]. In patients with suspected impairment, swallowing study should start with a lower amount—down to 3–5 mL according to the severity of the clinical situation.

Solids, modified fluids using thickening agents to achieve different consistencies, or natural foods such as pudding or crackers, are indicated to show a stricture, a solid-induced spasm or dysphagia, as well as for postoperative follow-up studies and to identify solid-induced abnormalities. The use of different consistencies—“nectar-like” (IDDSI Framework levels 1 or 2), honey-like” (IDDSI Framework level 3), or “spoon-thick” (IDDSI Framework level 4)—may reveal reduced risk of aspiration [66] and are extremely helpful in patients with an aspiration of only liquid boluses to assess further therapeutic and dietetic management [18]. The International Dysphagia Diet Standardisation Initiative IDDSI frame [67, 68] may be taken as a reference in defining the consistency given to the patient.

##### Best practice position statement 16

For VFSS, the choice of contrast material depends on national approval and is adapted to the specific procedure. Volumes and consistencies follow a standardised protocol which can be subjected to individual modification based on the patient’s abilities.

The professional performing a VFSS determines the entire exam protocol, following nationally defined medico-legal criteria of responsibility [3, 69, 70]. It is conceivable to start with a standardised protocol that is applicable to all patients and then move towards more personalised studies, which aim to elicit pathological swallowing patterns [18, 71, 72].

Logemann was the first to propose a uniform procedure where each patient was given two swallows of 2 mL liquid, 2 mL paste, and 1/4 cookie [73], but subsequently expanded the protocol to 1 mL, 3 mL, 5 mL, 10 mL, and cup drinking volumes [74]. In practice, there is a great variety of protocols depending on the clinician’s preference, patient population and clinical problems to be solved (e.g. [24, 75–78]).

The typical range of trials per bolus/consistency is 1–3. However, limited numbers of trials may underestimate aspiration risk [79]. For both diagnostic and therapeutic-guiding purposes, a sufficient number of swallows must be collected for analysis. For therapeutic-guiding purposes, it may be necessary to test multiple volumes and consistencies. It is an informal practice to start with the consistency considered least risky to the patient at the smallest volume, gradually increasing the volume before progressing to consistencies deemed most challenging.

Patients may be instructed to hold the bolus in their mouth and swallow when asked to (cued) or to swallow without any specific instruction (not cued). This results in differences in the patterns and timing of swallow onset [80, 81]. Specifically, the movement of the bolus to more distal locations in the pharynx at the moment of swallow onset is more frequently observed in noncued conditions [81]. This underscores the importance of recreating real-life conditions in specific situations.

#### Compensatory techniques and multidisciplinary team

##### Best practice position statement 17

Compensatory techniques can be part of the therapy-guiding VFSS exam to assess their effect on safety and efficiency during swallowing.

In addition to a standardised protocol consisting of repeated swallow trials using different viscosities and volumes, compensatory techniques may also be trialled. The primary purpose of compensatory manoeuvres is to improve safety and efficiency during swallowing. These manoeuvres have an immediate effect, aiming to compensate for abnormalities in the swallowing process. Techniques may include adjustments in body and head positioning, as well as bolus modification (e.g. changes in volume, consistency, temperature, and taste) [14]. The most commonly used compensatory head positioning techniques include the chin tuck, head turn, or head tilt. The chin tuck is recommended in case of unsafe swallowing [82, 83], whereas the head turn and head tilt can be applied in cases of asymmetry (e.g. laryngeal hemiparalysis) [84, 85].

In contrast, exercise techniques (e.g. oromotor exercises, Masako manoeuvre, or Shaker exercise) aim for permanent changes in the swallowing process by training specific musculature (e.g. tongue or orofacial musculature). This training can result in increased strength, coordination, and endurance of selected muscle groups [86]. As exercise techniques typically show effects over an extended period [87], they may not be useful during VFSS unless employed to confirm if a specific manoeuvre (e.g. Mendelsohn manoeuvre) is performed adequately.

##### Best practice position statement 18

The composition of the multidisciplinary healthcare team may differ across countries depending on national differences in education and the healthcare professionals involved in the management and care of people with dysphagia. The multidisciplinary healthcare team involved in performing a VFSS will be supervised by a radiologist or any other licensed physician, supported by a speech-language therapist or other trained allied health professional.

VFSS should be performed under the supervision of a radiologist or any other licensed physician trained in diagnostic radiology and the use of VFSS imaging equipment [69]. No VFSS should be performed in the absence of the medical supervisor. A therapy or radiology assistant/radiographer may support operating procedures related to the VFSS recordings and equipment. The assistant may be an allied health professional trained in supporting the VFSS assessment.

A speech-language therapist (SLT) will collaborate with radiological staff in the performance of VFSS. The SLT should have knowledge of the patient’s cultural and social background, as well as cognitive and communication status, to be taken into account during VFSS (and when developing a management plan based on VFSS findings). The SLT may recommend bolus textures and volumes, and optional rehabilitative swallow manoeuvres. An SLT may be involved in decisions around oral vs non-oral feeding, but is not qualified to determine the feeding route independently. An SLT is also not qualified to provide recommendations or interpretations about oesophageal functioning or abnormalities or to make any medical diagnosis. Formal training improves the agreement of VFSS interpretation across and within disciplines [88].

In addition to the listed professionals, the multidisciplinary healthcare team may consist of other members (e.g. dieticians, occupational therapists, physiotherapists, neurologists, or otolaryngologists) depending on national differences in education and healthcare professionals involved in the management and care of people with swallowing disorders [89].

## Summary

In this work, we developed position statements on best practices for performing videofluoroscopic swallowing studies in adult populations based on an international and interdisciplinary group of panellists representing two European societies. They provide a framework for the key technological concepts that should be taken into account when conducting this imaging study. The best possible compliance with technical requirements ensures quality assurance and improved patient care. A large consensus was reached to establish eighteen best practice position statements. These best practice position statements are not prescriptive guidelines. They may not be universally applicable, and their interpretation should consider specific clinical scenarios and resource availability.

## Abbreviations

ESGAR: European Society of Gastrointestinal and Abdominal Radiology
ESSD: European Society of Swallowing Disorders
SLT: Speech language therapist
VFSS: Videofluoroscopic swallowing study
